# Principal metabolic flux mode analysis

**DOI:** 10.1093/bioinformatics/bty049

**Published:** 2018-02-06

**Authors:** Sahely Bhadra, Peter Blomberg, Sandra Castillo, Juho Rousu

**Affiliations:** 1 Helsinki Institute for Information Technology HIIT, Department of Computer Science, Aalto University, Espoo, Finland; 2 Computer Science and Engineering, Indian Institute of Technology, Palakkad, India; 3 VTT Technical Research Centre of Finland Ltd, Espoo, Finland

## Abstract

**Motivation:**

In the analysis of metabolism, two distinct and complementary approaches are frequently used: Principal component analysis (PCA) and stoichiometric flux analysis. PCA is able to capture the main modes of variability in a set of experiments and does not make many prior assumptions about the data, but does not inherently take into account the flux mode structure of metabolism. Stoichiometric flux analysis methods, such as Flux Balance Analysis (FBA) and Elementary Mode Analysis, on the other hand, are able to capture the metabolic flux modes, however, they are primarily designed for the analysis of single samples at a time, and not best suited for exploratory analysis on a large sets of samples.

**Results:**

We propose a new methodology for the analysis of metabolism, called Principal Metabolic Flux Mode Analysis (PMFA), which marries the PCA and stoichiometric flux analysis approaches in an elegant regularized optimization framework. In short, the method incorporates a variance maximization objective form PCA coupled with a stoichiometric regularizer, which penalizes projections that are far from any flux modes of the network. For interpretability, we also introduce a sparse variant of PMFA that favours flux modes that contain a small number of reactions. Our experiments demonstrate the versatility and capabilities of our methodology. The proposed method can be applied to genome-scale metabolic network in efficient way as PMFA does not enumerate elementary modes. In addition, the method is more robust on out-of-steady steady-state experimental data than competing flux mode analysis approaches.

**Availability and implementation:**

Matlab software for PMFA and SPMFA and dataset used for experiments are available in https://github.com/aalto-ics-kepaco/PMFA.

**Supplementary information:**

Supplementary data are available at *Bioinformatics* online.

## 1 Introduction

Principal component analysis (PCA) is one of the most frequently applied statistical methods in systems biology (Barrett *et al.*, 2009; Ma and Dai, 2011; Yao *et al.*, 2012). PCA is used to reduce the dimensionality of the data while retaining most of the variation in the dataset. This reduction is done by identifying linear combinations of variables, called the principal components, that maximally explain the variation in the data. By using a few such components, each sample can be represented by relatively few variables compared to thousands of features. It also helps us to distinguish between biologically relevant variables and noise.

In the context of transcriptomics and fluxomics, PCA has been widely applied (Barrett *et al.*, 2009; Yao *et al.*, 2012), where a principal component (PC) identifies linear combinations of genes or enzymatic reactions whose activity changes explain a maximal fraction of variance within the set of samples under analysis. The main goals of PCA in fluxomic data are (i) to identify which parts of the metabolism retain the main variability in flux data and (ii) to relate them to the samples, *i.e.* behaviour of the organism for particular experimental condition.

However, in the context of fluxomics, PCA has a few limitations (Folch-Fortuny *et al.*, 2016): PCA considers reactions independently without considering any other structure or relationship among reactions, including stoichiometric relations implied by metabolic pathways. PCA simply extracts a set of reactions that are important to describe sample variance. Moreover, the principal components output by PCA are known to be generally dense, thus including most of the variables, which precludes their interpretation of pathways of any kind. It would be more useful for modelling and biological interpretation if the sample variance captured by the model could be expressed in terms of metabolic pathways or flux modes.

In this paper we propose a novel method to find metabolic flux modes that explains the variance in gene expression or fluxomic data collected from heterogeneous environmental conditions without requiring a fixed set of predefined pathways to be given. The proposed method is called as principal metabolic flux mode analysis (PMFA). Here each principal component, called *principal metabolic flux mode* (PMF), is found by selecting a set of reactions which represents a metabolic flux mode which is approximately in steady state and explains most of the data variability. In addition, we propose a sparse variant, called Sparse Principal Metabolic Flux Mode analysis (SPMFA), to further help the interpretation of the principal components.

Our method differs from existing methods in the literature such as Flux Balance Analysis (FBA) (Orth *et al.*, 2010) as well as more recent proposals as our method aims to explain the sample variability, while existing methods aim to extract flux modes that maximize an objective such as growth as in FBA, or a dominant flux modes active in a set of samples (Folch-Fortuny *et al.*, 2016; Stosch *et al.*, 2016). Related to our approach, Folch-Fortuny *et al.* (2015) has previously proposed multivariate curve resolution-alternating least squares to improve the biological interpretation of the principal components. Their method incorporates a few constraints such as non-negativity and selectivity when constructing the output. In addition, their method requires a fixed set of metabolic pathways to be defined as an initial step. Very recently, the Principal Elementary Mode Analysis (PEMA) was proposed (Folch-Fortuny *et al.*, 2016; Stosch *et al.*, 2016) where each component or principal elementary mode are selected from the complete set of elementary modes (EMs) (Pey and Planes, 2014) of the metabolic network such that the selected EMs are responsible for expression levels in a global data. This method needs to derive all possible elementary flux modes explicitly which prevents it to be applicable to genome-scale networks. Moreover, Folch-Fortuny *et al.* (2016, 2015) considered that all fluxes are in steady state, which restricts the applicability of the method in experiments containing transients, perturbations or high measurement noise (Baxter *et al.*, 2007).

The structure of this paper is as follows. The methods section describes the theory and development of a novel method to analysis fluxomic and gene expression data. The section includes the descriptions of data, means and algorithms by which the new method has been benchmarked. In the results section, we report a comparative study on the similarities and differences of PCA, SPCA, FBA, PMFA, SPMFA and PEMA. The study highlights four experiments. In the first experiment, we compare PMFA to PEMA in the retrieval of active elementary flux modes on a dataset for which the ground truth is known. In the second experiment, we study the effect of stoichiometric regularization on the fraction of test set variance explained by PMFA and alternative methods (PEMA, PCA) with Leave-One-Out (LOO) cross-validation. In the third experiment, SPMFA is used for the recovery of sparse flux modes from whole-genome *Saccharomyces cerevisiae* gene expression data where the performance is measured in terms of normalized variance captured. In the fourth experiment, elaborates on the biological findings obtained using SPMFA to analyze the variance in the mitochondrial subsystem of whole-genome *S.cerevisiae* metabolic network. We conclude the paper with discussion.

## 2 Materials and methods

### 2.1 Basic methods

Here we shortly review the existing basic methods for the analysis of fluxomic data.

*Principal component analysis:* We assume $X\in {\mathbb{R}}^{N\times N_{r}}$ be the data matrix of flux of *N* samples and *N_r_* reactions, with each entry corresponding to an estimated reaction rate for a particular reaction in a particular experiment. We assume throughout the paper that all variables have been centered to have zero empirical mean. The empirical covariance matrix is then given by $\Sigma =\frac{1}{N}X^{T}X$. Denoting $\Sigma_{1}=\Sigma$, the 1st principal component (PC) $w_{1}$ can be found by solving

(1) $$w_{1}=\underset{w\in {\mathbb{R}}^{N_{r}}}{\text{arg max}}w^{T}\Sigma_{1}w,\;\;\;\;\;\;\;\;\;\;\;\;\;\;\;\;s.t.\;||w|{|}_{2}=1$$

Above, $||w|{|}_{2}=\sqrt{w^{T}w}$ is the *l*_2_ norm of the vector w. The second PC can be found by applying Eq. (1) on updated the covariance matrix using deflation as $\Sigma_{2}=(1-w_{1}w_{1}^{T})\Sigma_{1}(1-w_{1}w_{1}^{T})$ (Mackey, 2009).

The weights, also called the loadings, of the principal component $w\in {\mathbb{R}}^{N_{r}}$ can be interpreted as the importance of reactions in explaining the variance in fluxomic data. The principal components are generally dense, containing most of the reactions of the metabolic network. Sparse PCA (Zou *et al.*, 2006) aims to increase the interpretabilty of PCA by finding principal components that have a small number of non-zero weights through solving the following optimization problem

(2) $$\underset{w}{\max}w^{T}\Sigma w-\lambda ||w|{|}_{1},\;\;\;\;\;\;\;\;\;\;\;\;\;\;s.t.\;||w|{|}_{2}=1$$

where *λ* is a user defined hyper-parameter which controls the degree of sparsity on PC. However, the principal components extracted by neither method represent metabolic flux modes, and will not in general adhere to thermodynamic constraints on reaction directions.

*Stoichiometric modelling:* The metabolic balance of the metabolic system is described using the exchange stoichiometric matrix $S\in {\mathbb{R}}^{N_{m}\times N_{r}}$ (Raman and Chandra, 2009) which contains transport reactions for inflow of nutrients and output flow of products, but does not contain any external metabolites (as they cannot be balanced). Rows of this matrix represent the *N_m_* internal metabolites, columns present the *N_r_* metabolic reactions including transport reactions and each element $S_{m,r}$ shows participation of the *m*th metabolite in the rth reaction: $S_{m,r}=1$ (or –1) indicates that reaction *r* produces (or consumes) the metabolite *m*. The value $S_{m,r}=0$ indicates metabolite *m* is not involved in the reaction *r*. For a flux vector w, Sw gives the change of metabolic concentration for all metabolites. The metabolic steady-state is assured by imposing a constraint Sw=0.

*Elementary modes:* The concept of an elementary mode (EM) (Pey and Planes, 2014; Ruppin *et al.*, 2010; Trinh *et al.*, 2009) is key for the analysis of metabolic networks. An EM is defined as a minimal set of cellular reactions able to operate at the steady-state, with each reaction weighted by the relative flux that they need to carry for the mode to function. An EM also satisfies the reaction directionality constraints arising from thermodynamics.

*Flux balance analysis (FBA):* FBA (Orth *et al.*, 2010) finds steady state flux modes maximizing objective function. Typically, FBA is done with an objective of maximizing biomass production by solving following optimization problem

(3) $$\underset{w}{\max}c^{T}w\;\;\;\;\;\;\;\;s.t.\;Sw=0\;\text{and}\;l\leq w\leq u$$

Here ***c****^T^* indicates the row from the stoichiometric matrix corresponding to biomass production.

### 2.2 Principal metabolic flux mode analysis (PMFA)

Here we describe our approach, Principal Metabolic Flux Mode Analysis (PMFA), that combines the PCA and stoichiometric modelling views of metabolism.

To obtain meaningful solutions of steady state flux distributions as PC loading one can impose two additional constraints in PCA formulation:

1. the weights associated with irreversible reactions should always be positive, i.e. $w_{ir}\geq 0$, where ir is an index of an irreversible reaction.
2. System is in a steady state, where the internal metabolite concentrations do not change, i.e. the metabolite producing and consuming fluxes cancel each other out: Sw=0.

Considering (1) and (2) the modified optimization problem for doing PCA with structural constraint is as following

(4) $$\begin{array}{l} \underset{w}{\max}\;\;\;\;\;\;\;\;w^{T}\Sigma w\\ \text{s}.\text{t}.\;\;\;\;\;\;\;\;\;\;\;Sw=0\;\;(\text{stoichiometric steady state})\\ w_{ir}\geq 0\;\;(\text{irreversible reactions can have only positive flux})\\ ||w|{|}_{2}=1 \end{array}$$

The constraint $||w|{|}_{2}=1$ restricts the spurious scaling up of the weights in the solution. Here, Sw=0 is a hard constraint and in practise imposes too much restriction, due to noise in the data, or when the data does not actually arise from steady-state conditions, e.g. given transients or perturbations of the fluxes during the experiment. Numerically one needs to solve a set of linear equation of size $N_{M}\times N_{R}$ which makes the problem also computationally hard to solve Eq. (4). Hence instead of considering this hard constraint on the PC loadings we introduce a soft constraint which penalizes the deviation from the steady state. Our aim is to find a flux which optimizes a combination of (1) maximal explained sample variance $w^{T}\Sigma w$ and (2) minimal deviation from a steady-state condition, expressed in the *l*_2_ norm: $||Sw-0|{|}_{2}^{2}=||Sw|{|}_{2}^{2}$. This entails solving the following optimization problem:

(5) $$\begin{array}{l} \underset{w}{\max}\;\;w^{T}\Sigma w-\lambda ||Sw|{|}_{2}^{2}\\ s.t.\;\;\;\;w_{ir}\geq 0\\ \;\;\;\;\;\;\;||w|{|}_{2}=1 \end{array}$$

Here *λ* imposes the degree of hardness of the steady-state constraint. For *λ* = 0 the Eq. (5) produces loadings similar to PCA with the exception of the reaction directionality constraint. The model will be henceforth denoted as $\mathrm{PMF}A^{\left(l_{2}\right)}$. If desirable, we can make our model to disregard reaction directionality simply by dropping the inequality constraints **w***_ir_* > 0. We denote this version of the method as rev-PMFA.

The *l*_2_ norm on Sw in Eq. (5) has the tendency to penalize large steady state deviations in individual metabolites, at the cost of favoring small deviations in many metabolites. This is probably the desired behaviour in case the data comes from conditions where there is no subsystems that is considerably farther from steady state than other parts of the system. In order to capture the opposite scenario, where a small subset of metabolites have large deviation from steady state, one can use *l*_1_ norm regularizer on Sw. The *l*_1_ norm regularizer $||Sw|{|}_{1}$ in Eq. (5) puts the emphasis of pushing most of the steady-state deviations to zero, whilst allowing a few outliers, metabolites that markedly deviate from steady state. Using *l*_1_ regularizer and a trade-off parameter *λ* we get to solve the following optimization problem:

(6) $$\begin{array}{l} \underset{w}{\max}\;\;\;\;\;\;w^{T}\Sigma w-\lambda ||Sw|{|}_{1}\\ s.t.\;\;\;\;\;\;\;\;\;w_{ir}\geq 0\\ ||w|{|}_{2}=1 \end{array}$$

Here *λ* imposes the degree of hardness of the steady-state constraint. Similarly to Eq. (5) for *λ* = 0 the Eq. (6) also produces loadings similar to PCA with selective non-negative constraint. The model will be hence forth denoted as $\mathrm{PMF}A^{\left(l_{1}\right)}$.

### 2.3 Sparse principal metabolic flux mode analysis

The above formulation of PCA with stoichiometric constraint still suffers from the fact that each principal component is typically a linear combination of all possible reaction activities, thus it is often difficult to interpret the results. This problem can be avoided by a variant of PMFA, the sparse principal metabolic flux mode analysis (SPMFA) using an *l*_1_ regularizer on w to produce modified principal components with sparse loadings.

(7) $$\begin{array}{l} \underset{w}{\max}\;\;\;\;\;\;w^{T}\Sigma w-\lambda ||Sw|{|}_{*}\\ s.t.\;\;\;\;\;\;\;\;\;\;w_{ir}\geq 0\\ \;\;\;\;\;\;\;\;\;\;\;||w|{|}_{1}=C \end{array}$$

where $||\cdot |{|}_{*}$ can be any of the *l*_2_ and *l*_1_ norm and *C* is a used defined hyper-parameter which controls the degree of sparsity in principal metabolic flux (PMF) loadings. Similarly to PMFA, Sparse PMFA can also be made to consider all reaction reversible by dropping the inequality constraints $w_{ir}\geq 0$. We call this variant rev-SPMFA.

### 2.4 Analysis of metabolic subsystems

One can apply our method to focus on variance within a subsystem of the whole metabolic network (e.g. central carbon metabolism, redox subsystem, lipid metabolism) by restricting the covariance matrix in objective function to the fluxes in the subsystem, while keeping the stoichiometric regularizer the same as before. Similarly, when some flux measurements are missing, one can change the covariance matrix in the objective function to exclude the measurements that are missing.

For example, to study the variation within the redox subsystem, let $X_{rdx}$ contain the columns of X corresponding to reactions containing redox co-factors, and let $w_{rdx}$ represent the corresponding part of w. We will consider $\Sigma_{rdx}=\frac{1}{N}X_{rdx}^{T}X_{rdx}$ for finding variance maximizing directions. Hence need to solve

(8) $$\begin{array}{l} \underset{w}{\max}\;\;\;\;\;\;\;w_{rdx}^{T}\Sigma_{rdx}w_{rdx}-\lambda ||Sw|{|}_{*}\\ s.t.\;\;\;\;\;\;\;\;\;w_{ir}\geq 0\text{and}||w|{|}_{2}=1 \end{array}$$

### 2.5 Algorithms

The objective function of Eq. (5) can be interpreted as difference of two differentiable convex functions. This type of optimization problem is known as Difference of Convex functions (DC) program. We used the convex-concave procedure (CPP), a local heuristic that utilizes the tools of convex optimization to find local optima of difference of convex functions (DC) programming problems (Lipp and Boyd, 2016). Using CCP method we solved Eq. (5) by solving following convex approximation (a Quadratic Program) in each iteration *t*:

(9) $$\begin{array}{l} w^{t+1}=\underset{w}{\text{arg}\;\text{min}}\frac{\lambda }{2}||Sw^{T}|{|}_{q}-w^{t^{T}}\Sigma_{E}w\\ \;\;\;s.t.\;\;\;\;\;\;\;w_{ir}\geq 0 \end{array}$$

followed by projecting $w^{t+1}$ on $||w|{|}_{p}=C$. The norms p,q∈{1,2} are chosen according to the desired model.

To find a good local optimum, we repeat the above optimization with different random starting points, and take the best local minimum as the solution. In our experiments we used 100 repetitions.

To obtain a *multi-factor* PMFA model, i.e. a model containing several PMFs jointly representing the data, we follow a approach similar to some PCA algorithms, namely the deflation of the covariance matrix. However, due to additional stoichiometric constraint here we deal with a sequence of non-orthogonal vectors, $\left[w_{1},\ldots ,w_{d}\right]$ hence we must take care to distinguish between the variance explained by a vector and the additional variance explained, given all previous vectors. We have used orthogonal projections for deflating the data matrix (Mackey, 2009). This also maintains the positive definiteness of covariance. For every iteration *d* + 1 we first transfer already found principal flux modes $W\in {\mathbb{R}}^{N_{R}\times d}$ to a set of orthogonal vectors, $\left\{q_{1},\ldots ,q_{d}\right\}$.

(10) $$q_{d}=\frac{(I-Q_{d-1}Q_{d-1}^{T})w_{d}}{\left||(I-Q_{d-1}Q_{d-1}^{T})w_{d}|\right|}$$

where $q_{1}=w_{1}$, and $q_{1},\ldots ,q_{d}$ form the columns of **Q***_d_*. $q_{1},\ldots ,q_{d}$ form an orthonormal basis for the space spanned by $w_{1},\ldots ,w_{d}$. Then the Schur complement deflation of covariance matrix is done by

(11) $$\Sigma_{d+1}=\Sigma_{d}-\frac{\Sigma_{d}q_{d}q_{d}^{T}\Sigma_{d}}{q_{d}^{T}\Sigma_{d}q_{d}}$$

## 3 Results

We report a comparative study on following methods.

- PCA: Principal component analysis as given by Eq. (1). PCA_*dir*_ denotes the PCA augmented with reaction directionality constraints.
- SPCA: Sparse PCA corresponding to Eq. (2). SPCA_*dir*_ is the SPCA augmented with reaction directionality constraints.
- FBA: Flux balance analysis with an objective of maximizing biomass production given by (3).
- PMFA: Principal Flux Mode Analysis as described in Section 2.2. $\mathrm{PMF}A^{\left(l_{2}\right)}$ denotes *l*_2_ regularization on the stoichiometric constraint Eq. (5) while $\mathrm{PMF}A^{\left(l_{1}\right)}$denotes *l*_1_ regularization on stoichiometric constraint Eq. (6).
- SPMFA: Sparse Principal Flux Mode Analysis as given by Eq. (7). Again, $\mathrm{SPMF}A^{\left(l_{2}\right)}$ denotes *l*_2_ regularization on stoichiometric constraint, while $\mathrm{SPMF}A^{\left(l_{1}\right)}$ denotes *l*_1_ regularization on stoichiometric constraint.
- Principal Elementary Mode Analysis (PEMA) (Folch-Fortuny *et al.*, 2016; Stosch *et al.*, 2016): It uses the set of EMs as the candidates for the PCs. It models the flux matrix X is as follows:
  (12) $$X=\Lambda P_{em}^{T}+E.$$

Above, **P***_em_* is the $N_{r}\times N_{f}$ principal elementary mode matrix, formed by a subset of *N_f_* EMs from the entire EM matrix; Λ is the $N\times N_{f}$ nonnegative weighting matrix; and **E** is the $N\times N_{r}$ residual matrix. **P***_em_* is found by iteratively selecting important EMs. We only used PEMA on small metabolic networks since as calculation of all EMs for genome-scale metabolic networks is impractically time consuming (Pey and Planes, 2014).

*Data centralization.* PCA, SPCA, PMFA and SPMFA aim at explaining the main variability in data using a few PCs. If the original variables have strongly different means and/or variances, the PCs may focus on explaining only the variables with the highest values and/or variances, disregarding the small variance associated with the rest of variables. Hence before applying all of them, we need to centralize the expression and fluxomic data.

*Selection of optimal level of regularization.* We selected the optimum levels of the regularization parameter *λ* for PMFA and SPMFA and level of sparsity for SPMFA by cross-validation maximizing the *fraction of sample variance explained* on test samples

$$\text{Fraction}\;\text{of}\;\text{variance}=\frac{w^{T}\Sigma w}{\text{Trace}(\Sigma )},$$

which is a classic measure used with PCA and related approaches. Above, w is the PC computed from the training data, and Σ is the co-variance matrix of the test sample. Leave-One-Out (LOO) cross-validation was used on smaller datasets and 5-fold cross-validation was used on the large whole genome dataset.

### 3.1 Datasets

*Pichia pastoris* simulation case study: We have used data generated by Stosch *et al.* (2016). It is based on the metabolic network of *Pichia pastoris*, which originates from Tortajada *et al.* (2010). It describes the central carbon metabolism of *P.pastoris* during growth on glucose, glycerol and methanol, comprising the Embden-Meyerhoff-Parnas pathway, citric acid cycle, penthose phosphate and fermentation pathways. It contains 45 compounds (36 of which are internal metabolites, which can be balanced for growth) and 44 reactions, yielding a total number of 98 EMs (Stosch *et al.*, 2016; Tortajada *et al.*, 2010). Flux data was generated simulating the growth of *P.pastoris* for twelve different cultivation conditions Stosch *et al.* (2016) by choosing appropriate sets of active EMs. Each active EM was drawn a random flux, and thus the flux distribution of each sample was a random linear combination of the fluxes of the active EMs.

Hence we can compare PMF identified by PMFA to the *ground truth* ‘active EMs’ that were used for data generation.This case study also enables the study of the impact of noise on the EMs identification and performance. For this study we add random Gaussian noise to fluxomic data, where noise variances are 2, 5, 10 and 20% of original values. From the flux data and the deviation reported in Supplementary Material of Quek *et al.* (2009) we observed that most the reported fluxes have deviation associated with it and the deviations are in range of 2–5% of their reported value along with few reactions with deviations even more than 12% of their value.

*Saccharomyces cerevisiae* experimental case study: A metabolic network for *S.cerevisiae* proposed by Hayakawa *et al.* (2015) and 13C isotopic tracer based fluxome data used in (Frick and Wittmann, 2005; Hayakawa *et al.*, 2015; Stosch *et al.*, 2016) was analyzed in this study. The network describes the central cytosolic and mitochondrial metabolism of *S.cerevisiae*, comprising glycolysis, the pentose phosphate pathway, anaplerotic carboxylation, fermentative pathways, the TCA cycle, malic enzyme and anabolic reactions from intermediary metabolites into anabolism (Stosch *et al.*, 2016). The network contains 42 compounds (30 of which are internal metabolites, which can be balanced for growth) and 47 reactions of which 39 are intracellular. The objective in this case study is to evaluate the performance of PMFA Eq. (5) on fluxome data and compare it with PEMA and PCA. For PEMA we have used 1182 EMs provided by Stosch *et al.* (2016).

*Saccharomyces cerevisiae* whole-genome metabolic network case study: The objective of experiment described in this section is to evaluate the performance of the proposed PMFA Eq. (5) and SPMFA Eq. (7) on whole-genome metabolic network in both steady-state and transient conditions. We used Yeast community model v. 7.5 (YCM 7.5), which contains 3494 reactions among 2220 compound and catalyzed by 909 genes.

The steady state transcriptomic data has been generated by Rintala *et al.* (2009) where *S.cerevisiae* grown in glucose-limited chemostat culture with 0, 0.5, 1.0, 2.8 or 20.9% oxygen in the inlet gas (D = 0.10/h, pH 5, 30°C) (Wiebe *et al.*, 2008). The normalized transcription dataset is available in the Gene Expression Omnibus (GEO) database (Barrett *et al.*, 2011) with the accession number GSE12442. It contains four steady state samples for 0, 0.5, 2.8 and 20.9% oxygen and six steady state samples for 1% oxygen. This dataset is combined with time-series transcriptomic data generated by Rintala *et al.* (2011) where time series analysis starting from two (1 and 20.9%) levels of oxygen provision. Seven time points at 0,0.2,3,8,16,24,72/79 hours from both time series and two biological replicates from each time point were analyzed. The microarray data can be accessed through GEO accession number GSE22832 (Barrett *et al.*, 2011).

We converted gene expression data to a expression level per reaction by with help of gene rules defined in metabolic network (Herrgård *et al.*, 2006; Jensen *et al.*, 2011). Gene rules are Boolean rules that determine the effect of the expression of regulatory genes on the activity of reactions in the metabolic network. Let us denote **X**^G^ as gene expression matrix with size $N\times N_{\text{G}}$ where N_G_ is number of genes and the **G**th column of **X**^G^, $x_{g}^{\text{G}}$ is the expression vector corresponding to gene *g*. Then,

- if gene association with reaction *r* is denoted as ‘*g*_1_ or *g*_2_’ then expression value for reaction *r*, i.e. $E_{r}=x_{g_{1}}^{\text{G}}+x_{g_{2}}^{\text{G}}$.
- otherwise if gene association with reaction *r* is denoted as ‘*g*_1_ and *g*_2_’ then expression value for reaction *r*, i.e. $E_{r}=\min(x_{g_{1}}^{\text{G}},x_{g_{2}}^{\text{G}})$.

### 3.2 Prediction of active EMs using PFMA

In our first experiment we evaluated the predictive performance the proposed PMFA and PEMA in correctly retrieving underlying active elementary flux modes. We used the *P.pastoris* simulation case study data, where the elementary flux modes that are part of the ground truth are known. For the evaluation, area under ROC curve (AUC) and area under precision recall curve (AUPR). The precision/recall metrics, widely used in information retrieval, is to assess how well the flux modes computed by PEMA and PMFA correlate with the ground truth active EMs. The PFM loadings are reported in Supplementary File PFMloading.ods in *PichiaPastorisResultAndAnalysis.zip*

For each PMF, we computed its correlation with respect to all 98 elementary flux modes of the *P.pastoris* metabolic network. We then sort the EMs in descending order of correlation and consider first i=1,…,98 EMs as the predicted EMs by the model. Precision and recall is then computed for each *i*, by considering ground truth active EMs within the first *i* EMs as true positives and other EMs with the top *i* as false positives. A precision/recall curve can be then plotted by taking the precision/recall values for all *i*s, in the order of the descending correlation in the sorted list. The AUPR is denoted as area under the precision recall curve and AUC is denoted as area under receiver operating characteristic curves (Hanley and McNeil, 1983).

In a PMFA model with *k* principal flux modes, to compute a precision-recall value for the model we considered the maximum correlation of an EM with any of the *k* principal flux modes as a final correlation of an EMs with the PMFA model. Then, we sorted all EMs according to descending order of their maximum correlations. With PEMA model we used an analogous approach: for a PEMA model containing *k* EMs, for each *i* we included the top *i* correlated EMs (according to the maximum correlation of EMs with any of the *k* EM’s chosen by PEMA) as the models prediction and used those for computing the precision/recall values for each i=1,…,98.

**Fig. 1.**
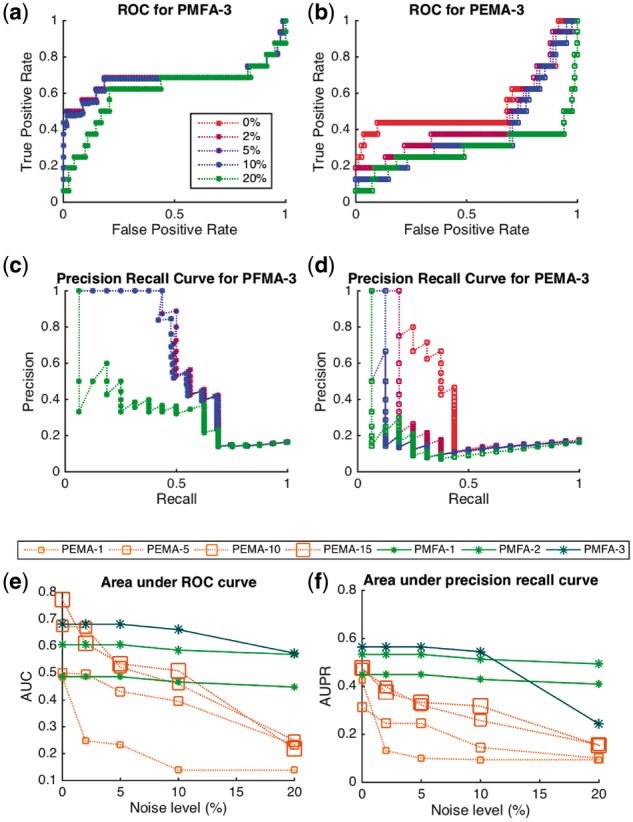
The graph the first 3 components of models and shows (**a**) ROC for PMFA, (**b**) ROC for PEMA,(**c**) and precision-recall curves for PMFA and (**d**) PEMA for different noise levels. (**e**) and (**f**) plots respectively AUC and AUPR values obtained by different models for different noise levels

Figure 1 shows (a–b) Receiver operating characteristic curves (ROC), (c–d) precision-recall curves and (e) total AUC and (f) total AUPR achieved by the different models for different amount of additional noise. It shows that PMFA is robust with respect to noise in the fluxomic data, with both AUPR and AUC metrics only slowly decreasing as a function of increasing noise, until noise level of 10%. In this regime, adding more factors to PMFA models also increases performance monotonically both in AUC and AUPR metrics, showing that the additional factors recover EMs that were not captured by the first factor. In the high noise regime (>10%) we observe that the performance of the 3-factor PMFA model drops suggesting that the last factor likely starts to capture noise.

In the noise free case, PEMA performs comparatively to PMFA, especially in terms of the AUC metric and when using a high enough number of factors in the model. However, the performance of PEMA deteriorates quickly upon increased noise. The decrease of performance is particularly apparent in the AUPR metric.

### 3.3 Explaining test set variance with PMFA

In this experiment we focused on the ability of PMFA to explain variance on data in a predictive setting, that is, on new data that has not been used for model estimation. We focused on the amount of variance explained in the test set in a Leave-One-Out (LOO) cross-validation setting.

**Fig. 2.**
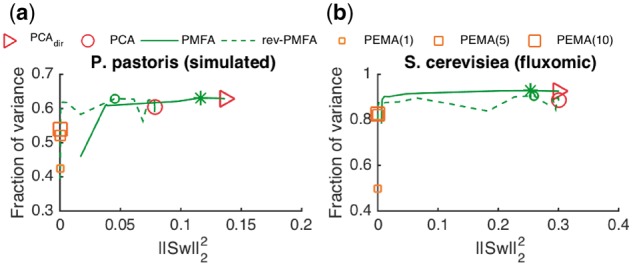
Depicted is for two fluxomic datasets the fraction of variance on test data in LOO setting as a function of deviation from steady state ($||Sw|{|}_{2}^{2}$) captured by PCA, directional PCA (PCA_*dir*_), 1-, 5- and 10-factor PEMA, as well as PMFA and rev-PMFA using different amount of Stoicihiometric regularization. The markers ‘*’ and ‘*o*’ indicate the optimal level of regularization for PMFA and rev-PMFA

We studied the effect of stoichiometric regularization ($\lambda ||Sw|{|}_{2}^{2}$) on the fraction of sample variance captured by PMFA and alternative models (PEMA, PCA). Figure 2 shows the fraction of sample variance explained by the first PMFs and PCs as a function of deviation from steady state ($||Sw|{|}_{2}^{2}$) in test data of two fluxomic datasets (*S.cerevisiae* and *P.pastoris*). The deviation from the steady-state is controlled by the regularization parameter λ≥0: high values of *λ* give low deviation from steady-state and vice-versa.

In particular on the fluxomic datasets, relatively heavy regularization can be applied without decrease of variance explained, indicating that the data can be well explained by steady-state flux modes.

By change of the regularization parameter *λ*, the statistics of PMFA exhibit a continuous transition from fully steady state flux modes ($||Sw|{|}_{2}^{2}=0$) to the *PCA_dir_*, i.e. PCA augmented with reaction directionality constraints (corresponding to *λ* = 0 in PMFA). The transition for rev-PMFA is not as smooth as PMFA with the directionality constraint. It is apparent that the directionality constraint increases the stability of PMFA without reducing much explained variance on test data.

Compared to PEMA, The fraction of variance explained the first PMF from rev-PMFA is higher than 1-, 5- and 10-factor PEMA regardless of the amount of stoichiometric regularization or application of the directionality constraints. The amount of variance explained by the first PMF from PMFA is also much higher than 1-factor PEMA even with high Stoichiometric regularization, while the 5- and 10-factor PEMA reach the level of PMFA for both datasets.

**Fig. 3.**
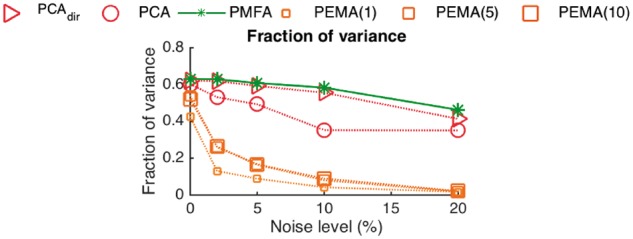
Depicted is for the *P.pastoris* simulated dataset the fraction of variance on test data in LOO setting as a function of additional noise level captured by PCA, PCA_*dir*_ 1-, 5- and 10-factor PEMA, as well as PMFA (with optimum regularization parameter)

Figure 3 shows the explained fraction of variance on test data in a Leave-One-Out (LOO) cross-validation setting, where both test and training data is contaminated with various amount of the noise. The test set variance captured by first component of PMFA only very slightly decreases upon increasing noise. In contrast, the test set variance captured by PEMA drops considerably when the noise level increases. Higher order PEMA models are here somewhat more resistant than the 1-factor PEMA but still not competitive with PMFA. In addition, we note that PCA is not able to explain test set variance as well as PMFA regardless of the noise level. To understand this result, we note that within the training set, by definition we expect PCA to explain the variance the best. However, when analyzing new data not seen in the training phase, the stoichiometric information used by PMFA helps to attain a better predictive performance.

### 3.4 Recovery of sparse flux modes from full genome data by SPMFA

In this experiment, we evaluated the Sparse Principal Metabolic Flux Mode Analysis, SPMFA, in discovery of sparse flux modes, i.e. only few reactions with non-zero coefficients. We focus on the full genome data, i.e. all steady-state and transient samples of *S.cerevisiae* containing a total of 3494 reactions for, making dense principal components and flux modes difficult to interpret. The SPFM loadings along with the amount of inter-cellular metabolites produced or consumed by SPFM for various degree of steady state constraints are reported in Supplementary File SPFM-geneexpression.ods in *SPFMoxygenseriesResultandAnalysis.zip*.To quantify the fraction of explained variance normalized by the complexity of the extracted flux mode, we measure the *normalized fraction of variance*, calculated as

$$\text{Normalized}\;\text{variance}=\frac{\text{Fraction}\;\text{of}\;\text{variance}\;\text{explained}}{||w|{|}_{0}/N_{r}}.$$

Above, $||w|{|}_{0}$ denotes the *l*_0_ norm, i.e. the cardinality of non-zero elements of wFigure 4 shows variance (left) and normalized variance (right) as the function of deviation from steady state ($||Sw|{|}_{2}^{2}$).

**Fig. 4.**
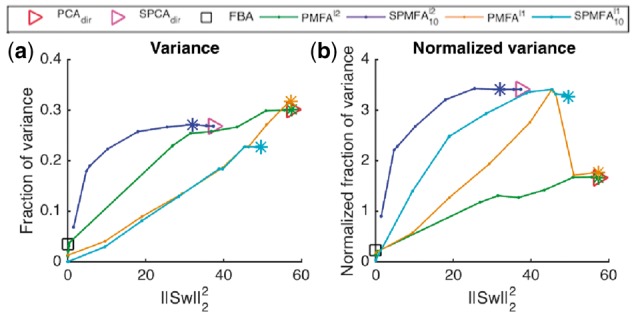
Variance (left) and normalized variance (right) on test data in 5 fold cross validation setting as a function of steady state deviation ($||Sw|{|}_{2}^{2}$) on the whole genome gene expression data (containing both steady-state and transient samples) for PMFA SPMFA and FBA. The markers ‘*’ indicate the optimal level of regularization

At the maximum, PMFA captures slightly more explained variance than SPMFA at (Fig. 4, left). Correspondingly, SPMFA is vastly more effective in capturing normalized variance, achieving more than double the rate of PMFA at any level of deviation from steady state (Fig. 4, right). SPMFA statistics can be seen to smoothly approach the (directional) sparse PCA statistics when the deviation from steady-state is let to increase.

The variant $\mathrm{SPMF}A^{\left(l_{1}\right)}$ which is regularized by the *l*_1_ stoichiometric regularizer ($||Sw|{|}_{1}$), also exhibit a smooth transition, but captures less variance at the maximum, albeit the fraction of normalized variance captured is similar to SPMFA. PMFA$\mathrm{PMF}A^{\left(l_{1}\right)}$ exhibits a phase change, following PMFA at high steady state distances (small *λ*) but switching to SPMFA regime as regularization is increased. This reflects the fact that with small *λ* the model is not yet sparse but sparsity quickly emerges once *λ* is increased.

It is notable that on this large heterogeneous dataset, all methods fail to capture meaningful amounts of normalized sample variance in the vicinity the steady state ($||Sw|{|}_{2}^{2}=0$). This is also true for FBA, which we have included as a comparison (maximum biomass production as the FBA objective). The FBA solution is sparse but the fraction of variance captured is very small, causing as the normalized variance captured by FBA to be small compared to SPMFA solution when the stoichiometric regularization is relaxed. This illustrates the importance of being able to relax the steady-state assumption when analyzing real-world experiments.

### 3.5 Analysis of SPMFA on *S.cerevisiae* oxygen series gene expression dataset

In this experiment, we analyze the Principal Metabolic Flux Modes found by SPMFA when analyzing the variance in the subsystem composed of the reactions in the mitochondrion of the *S.cerevisiae* whole-genome network. The availability of oxygen limits the amount of ATP the cell can generate. Oxidative phosphorylation occurs in the mitochondrion. The mitochondria are unique organelles that replicate, transcribe enzymes, and possibly adapt to changes in oxygenation level somewhat independently from the rest of the organism. Therefore, we elected to study this organelle in more detail.

We used the method described in Section 2.4 for the analysis, where the covariance matrix is obtained from the 166 mitochondrial reactions in the combined data consisting of the time-series and steady-state samples. For the stoichiometric regularizer the stoichiometric matrix of the whole-genome network of a total of 3494 reactions was used. We use regularization level *λ* = 1 as it gave the most interpretable results. The PFM corresponding to all mitocondrion reactions and metabolites changes due to this flux are reported in Supplementary Tables S2–S4 and Table S5 in Supplementary File *PMFAsup.pdf*.

**Fig. 5.**
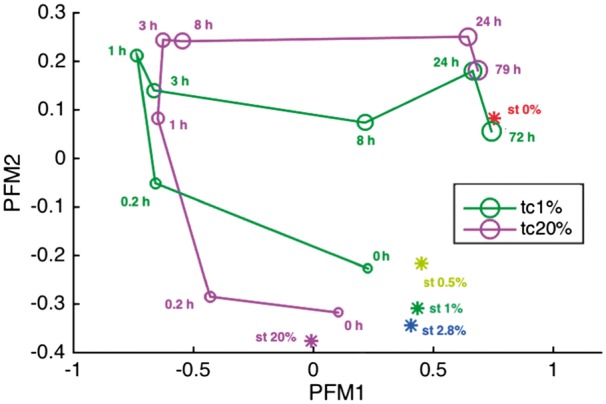
The correlation of expression data for corresponding samples with first two SPMFs at expert chosen *λ*. Here we have considered PMFA with *L*_2_ constrain on Sw on all samples but only mitochondrion reactions of *S.cerevisiae* oxygen series gene expression dataset

Figure 5 depicts the scores of the samples in the first two PMFs. The two components clustered the initial (0 h) time-series samples with the steady-state samples with oxygenation, the early time-series samples (0.2–3 h) together as well as the late time-series samples (24–79 h) with the steady-state sample without oxygenation.

1st PMF correlated the best with late time-series samples, where the system approached the new anaerobic steady state, as well as the anaerobic steady state sample. In addition, the 1st PMF correlates negatively with the early time-series samples (0.2–3 h) representing a state shortly after the loss of oxygen. The 2nd PMF discriminates among samples with different oxygen level in the environment, with samples correlating with the 2nd PMF the better the less oxygen is available. This is evident in the monotonic decreasing correlation of the steady-state samples based on the oxygen level, as well as the generally increasing correlation of the time-series samples as a function of time (and decreasing oxygen).

Six metabolic pathways emerged upon closer inspection of the individual reactions associated with 1st PMF and 2nd PMF. Two of these pathways were associated with the 1st PMF while four pathways were associated with the 2nd PMF. The main reactions and their loadings are shown in Table 1. The pathways are denoted by the following letters: A. Malic pathway, B. Acetaldehyde pathway, C. Malate shuttle, D. Oxidative phosphorylation, E. Tetrahydrofolate pathway and F. ATP pathway.

**Table 1.** The loadings for few selected reactions corresponding to the first and second PMFs while taking *l*_2_ regularization on amount of metabolites produced or consumed

Pathways	Reactions	1st PMF	2nd PMF
A	Malic enzyme (NAD)	1.40	0
A	Malic enzyme (NADP)	1.65	0
A	Acetolactate synthase	1.72	0
A	Pyruvate transport	1.90	0
A	Malate transport	2.58	0.83
B	Hydroxymethylglutaryl CoA synthase	0	0.52
B	Acetyl-CoA synthetase	0	0.83
B	Acetaldehyde dehydrogenase (NADP)	0.10	0.83
C	Aspartate-glutamate transport	0	0.83
C	Glutamate transport	0	0.83
C	Malate dehydrogenase	0.71	0.83
C	Oxoglutarate/malate exchange	0.96	0.83
D	Ubiquinol: ferricytochrome c reductase	0	0.83
D	Ferrocytochrome-c: oxygen oxidoreductase	0	0.83
D	ATP synthase	0.16	0.83
D	Succinate dehydrogenase (ubiquinone-6)	0.52	0.83
E	Glycine-cleavage complex (lipoylprotein)	0	0.65
E	Glycine hydroxymethyltransferase	−0.34	0.66
E	Glycine-cleavage complex (lipoylprotein)	0.14	0.70
E	MethenylTHF cyclohydrolase	−0.29	0.71
E	Glycine-cleavage complex (lipoylprotein)	0	0.83
E	MethyleneTHF dehydrogenase (NADP)	−0.56	0.83
E	Methionyl-tRNA formyltransferase	1.12	0.83
F	Acetyl-CoA acetyltransferase	1.42	0
F	Oxoglutarate dehydrogenase (lipoamide)	1.20	0.83
F	Oxoglutarate dehydrogenase	1.26	0.83
F	Succinyl-CoA: acetate CoA transferase	−1.98	0.12
F	ADP/ATP transport	2.23	−0.83
F	Succinate transport	2.48	0.10
F	2-Oxoglutarate transport	2.62	0.38
	Total variance captured (%)	25.20	26.57
	Total steady state deviation $||Sw|{|}_{2}^{2}$	22.15	4.01

*Note*: Pathways can be called as A. Malic pathway, B. Acetaldehyde pathway, C. Malate shuttle, D. Oxidative phosphorylation, E. Tetrahydrofolate pathway and F. ATP pathway.

The Malic pathway (A) associated with the 1st PMF consisted of malate import, dehydrogenation to produce NADPH and/or NADH, pyruvate export and acetolactate synthesis from pyruvate. The ATP pathway (F) associated with the 1st PMF included oxoglutarate import, TCA cycle reactions from oxoglutarate to succinyl-CoA, succinate export and two means to extract the ATP-equivalent stored in succinyl-CoA. The negative loading of Succinyl-CoA: acetate CoA transferase may indicate a switch from this reaction to other reactions generating ATP more explicitly. This hypothesis is supported by the full set of reactions in the Supplementary *PMFAsup.pdf*, Supplementary Table S2–S4. However, Succinyl-CoA: acetate CoA transferase produce a non-negligible amount of acetyl-CoA, which is subsequently converted to acetoacetyl-CoA by Acetyl-CoA acetyltransferase. The ATP pathway also included the direct transport of ATP between the cytosol and the mitochondrion. The Malic pathway’s capability to provide the mitochondrion with reducing equivalents in the form of NADPH and NADH, and the ATP pathway’s capability to provide the mitochondrion with ATP are apparently captured by the PMF.

The Acetaldehyde pathway (B) associated with the 2nd PMF represents the conversion of acetaldehyde to acetate with the generation of NADPH, and the sequestration of the formed acetate to acetyl-CoA and further to hydroxymethylglutaryl-CoA, an intermediate in the mevalonate and the ketogenesis pathways. The 2nd PMF contained the Malate shuttle for generating mitochondrial NADPH. The 2nd PMF also contained the reactions for the electron transport chain and oxidative phosphorylation (D), possibly for the removal of residual oxygen. In (E), a pathway catabolizing pyruvate via Glycine hydroxymethyltransferase, the Glycine cleavage complex, Methylene-THF dehydrogenase and Methenyl-THF cyclohydrolase is captured. The Tetrahydrofolate pathway (E) ended with Methionyl-tRNA formyltransferase, thus producing one NADH and one NADPH per pyruvate catabolized. The four pathways associated with the 2nd PMF appear to capture the generation of mitochondrial NADPH, a vital cofactor for metabolic adaptation by biosynthesis.

## 4 Discussion

In this paper we have proposed a novel method for the analysis of metabolic networks, called the Principal Metabolic Flux Analysis, PMFA, through the combination of stoichiometric flux analysis and principal component analysis, finds flux modes that explain most of the variation in fluxes in a set of samples. Unlike most stoichiometric modeling methods, PMFA is not tied to the steady-state assumption, but can automatically adapt—by the change of a single regularization parameter—to deviations from the stoichiometric steady-state, whether they are due to measurement errors, biological variation or other causes. Our experiments showed that the method is more robust to the steady-state violations than competing approaches, and can compactly capture the variation in the data by a few factors. For the analysis of whole-genome metabolic networks, we further proposed Sparse Principal Flux Mode Analysis, SPMFA that allows us to discover flux modes with a small fraction of reactions activated, thus could be interpreted as pathways. Our experiments showed that our methods are more efficient in capturing the variance in sets of experiments than methods based on elementary flux mode analysis or flux balance analysis. The efficient Concave Convex Procedure optimization allows the method to scale up to whole-genome models unlike methods based on search in the space of elementary flux modes.

Analysis of cultivation data on the whole-genome metabolic network of *S.cerevisiae* showed that PMFA was able to identify six mitochondrial pathways responsive to changes in oxygen availability. In addition, the analysis grouped these pathways in easily interpretable pathways.

The Malic pathway’s capability to provide the mitochondrion with reducing equivalents in the form of NADPH and NADH, and the ATP pathway’s capability to provide the mitochondrion with ATP were apparently captured by the 1st PMF. The four pathways associated with the 2nd PMF appeared to capture the generation of mitochondrial NADPH, a vital cofactor for metabolic adaptation by biosynthesis.

## Supplementary Material

Supplementary Data
