# Supplementary material for: Principal metabolic flux mode analysis
Source: Bioinformatics. 2018 Feb 6;34(14):2409–17. doi: 10.1093/bioinformatics/bty049 (PMC6041797; doi:10.1093/bioinformatics/bty049)
Supplement: Supplementary Data [file bty049_supp.zip › bty049-suppl_data/PMFAsup.pdf]

Table 2. Tables shows the fluxes for mitochondrion reactions corresponding to the first and second PMFs while taking l_2 regularisation on amount of metabolites produced or consumed.

Reaction Names	1st PMF	2nd PMF
AKG transporter, mitochonrial	2.62	0.38
malate transport	2.58	0.83
succinate transport	2.48	0.10
glutamyl-tRNA synthetase	2.39	-
L-lactate transport	2.34	-0.41
ADP/ATP transporter	2.23	-0.83
succinyl-CoA:acetate CoA transferase	-1.98	0.12
pyruvate transport	1.90	-
NADH dehydrogenase, cytosolic/mitochondrial	1.87	-
acetolactate synthase	1.72	-
malic enzyme (NADP)	1.65	-
glycine-cleavage complex (lipoamide)	1.42	0.83
methionyl-tRNA formyltransferase	1.12	0.83
ferrocytochrome-c:oxygen oxidoreductase	-	0.83
oxoglutarate dehydrogenase (dihydrolipoamide S-succinyltransferase)	1.26	0.83
methylenetetrahydrofolate dehydrogenase (NADP)	-0.56	0.83
oxoglutarate dehydrogenase (lipoamide)	1.20	0.83
aldehyde dehydrogenase (acetylaldehyde, NADP)	0.10	0.83
succinate dehydrogenase (ubiquinone-6)	0.52	0.83
citrate to cis-aconitate(3-)	0.30	0.83
ATP synthase	0.16	0.83
ubiquinol:ferricytochrome c reductase	-	0.83
adenylate kinase (GTP)	0.34	0.83
oxoglutarate/malate exchange	0.96	0.83
L-glutamate transport	-	0.83
carnitine O-acetyltransferase	-	0.83
aspartate-glutamate transporter	-	0.83
acetyl-CoA synthetase	-	0.83
phosphate transport	1.24	0.83
glycine-cleavage complex (lipoylprotein)	-	0.83
(S)-lactate:ferricytochrome-c 2-oxidoreductase	-	0.83
malate dehydrogenase	0.71	0.83
citrate transport	0.54	0.83
2-aceto-2-hydroxybutanoate synthase	1.58	0.07
cis-aconitate(3-) to isocitrate	0.23	0.79
nicotinate-nucleotide diphosphorylase (carboxylating)	1.48	-
acetyl-CoA C-acetyltransferase	1.42	-
2-deoxy-D-arabino-heptulosonate 7-phosphate synthetase	1.41	-
malic enzyme (NAD)	1.40	-
aldehyde dehydrogenase (indole-3-acetaldehyde, NADP)	0.19	0.72
methenyltetrahydrifikate cyclohydrolase	-0.29	0.71
thioredoxin reductase (NADPH)	1.36	0.57
glycine-cleavage complex (lipoylprotein)	0.14	0.70
thiamine diphosphate transport	1.33	-
succinate-fumarate transport	0.13	0.68
2-methylcitrate dehydratase	1.31	-0.09
glycine hydroxymethyltransferase	-0.34	0.66
glycine-cleavage complex (lipoylprotein)	-	0.65
L-4-hydroxyglutamate semialdehyde dehydrogenase	1.26	-0.11
riboflavin kinase	-	0.63
GTP/GDP translocase	0.33	0.62
homoacontinate hydratase	1.15	-0.10
1-pyrroline-5-carboxylate dehydrogenase	1.12	-0.28
L-1-pyrroline-3-hydroxy-5-carboxylate dehydrogenase	1.04	-0.53
inorganic diphosphatase	0.82	0.54
hydroxymethylglutaryl CoA synthase	-	0.52
fadH2 transport	0.98	0.21
citrate transport	0.47	0.50
phenylalanyl-tRNA synthetase	0.97	-
2-isopropylmalate synthase	0.20	0.44
aldehyde dehydrogenase (2-methylbutanol, NAD)	0.38	0.43
aldehyde dehydrogenase (isobutyl alcohol, NAD)	0.38	0.43
aldehyde dehydrogenase (isoamyl alcohol, NAD)	0.38	0.43
aldehyde dehydrogenase (2-phenylethanol, NAD)	0.38	0.43
ornithine transport	0.24	0.43
leucyl-tRNA synthetase	0.83	-
dihydropteroate synthase	-	0.43
coenzyme A transport	-	0.43
proline oxidase (NAD)	0.82	-
S-adenosyl-L-methionine transport	-0.53	0.42
asparaginyl-tRNA synthetase, miotchondrial	0.78	-
3-methyl-2-oxobutanoate hydroxymethyltransferase	0.77	0.21
dihydroneopterin aldolase	-	0.40
histidyl-tRNA synthetase	0.76	0.06
2-oxoadipate and 2-oxoglutarate transport	0.74	-0.11
guanosine phosphorylase	0.73	-0.14
oxaloacetate transport	0.71	-
ornithine transacetylase	-	0.37

Table 3. Continue....Tables 2

Reaction Names	1st PMF	2nd PMF
NAD kinase	-	0.36
hydrogen peroxide reductase (thioredoxin)	0.70	0.30
itaconate-CoA ligase (ADP-forming)	0.60	-0.34
acetylglutamate kinase	0.64	0.08
dihydrofolate reductase	-	0.33
NADH:ubiquinone oxidoreductase	0.60	-
adenylate kinase	0.59	0.22
aconitase	-0.59	-
N-acetyl-g-glutamyl-phosphate reductase	0.57	0.20
formate-tetrahydrofolate ligase	0.03	-0.30
glycerol-3-phosphate dehydrogenase (NAD)	0.56	-
ketol-acid reductoisomerase (2-aceto-2-hydroxybutanoate)	-	0.29
Aminobutyraldehyde dehydrogenase	-	0.28
aldehyde dehydrogenase (tryptophol, NAD)	0.37	0.26
malonyl-CoA-ACP transacylase	0.51	0.18
acetohydroxy acid isomeroreductase	-	0.26
acetyl-CoA ACP transacylase	0.49	0.11
mitochondrial alcohol dehydrogenase	0.38	0.25
glycerol-3-phosphate dehydrogenase (fad)	0.47	-
methylisocitrate lyase	0.46	-
acetyl-Coa carboxylase	-	0.24
tetrahydrofolate aminomethyltransferase	0.16	0.23
2-methylcitrate synthase	-	0.22
hexaprenyldihydroxybenzoate methyltransferase	-	0.21
threonyl-tRNA synthetase	0.40	-
2-hexaprenyl-6-methoxy-1,4-benzoquinone methyltransferase	-	0.20
UTP/UMP antiport	0.34	-
CTP/CMP antiport	0.34	-
citrate transport	0.33	-0.18
protoporphyrinogen oxidase	0.34	-
fumarase	-0.24	0.17
CTP transport	0.20	0.17
2-hexaprenyl-6-methoxyphenol monooxygenase	-	0.17
arginyl-tRNA synthetase	0.29	0.17
quinone oxidoreductase	-	0.17
UTP transport	0.14	0.16
succinate-CoA ligase (ADP-forming)	0.31	0.07
Heme O synthase	-	0.14
glutathione oxidoreductase	0.27	-
Aspartyl-tRNA synthetase	0.27	-
aldehyde dehydrogenase (acetylaldehyde, NAD)	-	0.14
glutathione peroxidase, mitochondria	-0.25	-0.09
citrate synthase	-	0.12
S-adenosyl-L-methionine:3-hexaprenyl-4,5-dihydroxylate O-methyltransferase	-	0.10
L-erythro-4-hydroxyglutamate:2-oxoglutarate aminotransferase	0.20	-
isocitrate dehydrogenase (NAD+)	0.18	-
heme O monooxygenase	0.17	-
hydroxybenzoate octaprenyltransferase	-	0.08
glycine-cleavage system (lipoamide)	0.16	-
tryptophanyl-tRNA synthetase	0.13	-
tyrosyl-tRNA synthetase	-	0.06
isoleucyl-tRNA synthetase	-	0.05
NAD transport	0.10	-
fumarate reductase	0.10	0.05
NAD transport	0.10	-
(R)-lactate:ferricytochrome-c 2-oxidoreductase	0.07	-
carnithine-acetylcarnithine carrier	0.06	-
dihydropteroate synthase	-	-
homoisocitrate dehydrogenase	0.03	-
(R)-lactate:ferricytochrome-c 2-oxidoreductase	-	-
NAD transport	-	-
N-acteylglutamate synthase	-	-
hydroxyacylglutathione hydrolase	-	-
2-oxo-4-methyl-3-carboxypentanoate decarboxylation	-	-
NAD transport	-	-
L-threonine deaminase	-	-
homocitrate synthase	-	-
acetyl-CoA hydrolase	-	-
dephospho-CoA kinase	-	-
trans-pentaprenyltranstransferase	-	-
glycine cleavage system	-	-
aldehyde dehydrogenase (indole-3-acetaldehyde, NAD)	-	-

Table 4. Continue....Tables 2

Reaction Names	1st PMF	2nd PMF
dehydrogenase	-	-
NADH kinase mitochondrial	-	-
deoxyhypusine synthase, cytosolic/mitochondrial	-	-
ferrocytochrome-c:hydrogen-peroxide oxidoreductase	-	-
acyl carrier protein synthase	-	-
ferrochelatase	-	-
NAPRtase	-	-
dihydroxy-acid dehydratase (2,3-dihydroxy-3-methylpentanoate)	-	-
2-amino-4-hydroxy-6-hydroxymethyldihydropteridine diphosphokinase	-	-
dihydroxy-acid dehydratase (2,3-dihydroxy-3-methylbutanoate)	-	-
5-aminolevulinate synthase	-	-
lysyl-tRNA synthetase	-	-
glycine-cleavage complex (lipoamide)	-	-
isocitrate dehydrogenase	-	-
methionyl-tRNA synthetase	-	-
valyl-tRNA synthetase	-	-

Table 5. Tables shows the mitochondrion metabolites produced or consumed by the first and second PMFs while taking l_2 regularisation on amount of metabolites produced or consumed.

Reaction Names	1st PMF	2nd PMF
ATP [mitochondrion]	-1.10	-
carbon dioxide [mitochondrion]	0.88	0.05
diphosphate [mitochondrion]	0.80	-
AMP [mitochondrion]	0.59	-
H2O [mitochondrion]	-0.58	-
acetyl-CoA [mitochondrion]	-0.55	0.04
ADP [mitochondrion]	0.49	-
H+ [mitochondrion]	0.40	-
NADH [mitochondrion]	0.36	-0.04
NAD [mitochondrion]	-0.34	-
ubiquinol-6 [mitochondrion]	0.30	-
ubiquinone-6 [mitochondrion]	-0.30	-
succinyl-CoA [mitochondrion]	0.29	0.06
Glu-tRNA(Glu) [mitochondrion]	0.24	-
tRNA(Glu), mitchondrial [mitochondrion]	-0.24	-
(S)-lactate [mitochondrion]	0.23	-0.04
acetate [mitochondrion]	0.21	-
erythro-4-hydroxy-L-glutamic acid [mitochondrion]	0.20	-0.04
THF [mitochondrion]	0.19	0.03
phosphoenolpyruvate [mitochondrion]	-0.17	-
2-acetyllactic acid [mitochondrion]	0.17	-0.03
(S)-2-acetyl-2-hydroxybutanoate [mitochondrion]	0.16	-
citrate [mitochondrion]	-0.16	-
2-oxobutanoate [mitochondrion]	-0.15	-
nicotinic acid D-ribonucleotide [mitochondrion]	0.15	-
acetoacetyl-CoA [mitochondrion]	0.14	-0.05
PRPP [mitochondrion]	-0.14	-
quinolinate [mitochondrion]	-0.14	-
10-formyl-THF [mitochondrion]	-0.14	-0.04
2-oxoglutarate [mitochondrion]	0.14	-0.03
TDP [mitochondrion]	0.13	-
7-phospho-2-dehydro-3-deoxy-D-arabino-heptonic acid [mitochondrion]	0.13	-
D-erythrose 4-phosphate [mitochondrion]	-0.13	-
homocitrate [mitochondrion]	-0.13	-
L-glutamate [mitochondrion]	-0.13	-
L-4-hydroxyglutamic semialdehyde [mitochondrion]	-0.13	0.04
oxygen [mitochondrion]	-0.13	-0.03
oxaloacetate [mitochondrion]	0.12	-0.03
coenzyme A [mitochondrion]	0.12	-
homoisocitrate [mitochondrion]	0.11	-
fMet-tRNA(fMet) [mitochondrion]	0.11	0.08
Met-tRNA(Met) [mitochondrion]	-0.11	-0.08
glutathione [mitochondrion]	0.11	-
(2S,3R)-3-hydroxybutane-1,2,3-tricarboxylic acid [mitochondrion]	-0.10	-
1-pyrroline-3-hydroxy-5-carboxylic acid [mitochondrion]	-0.10	0.03
(S)-malate [mitochondrion]	-0.10	-
ACP1 [mitochondrion]	-0.10	-
oxaloacetate [extracellular]	-	-0.10
succinate [mitochondrion]	0.10	-
FAD [mitochondrion]	-0.10	-
Phe-tRNA(Phe) [mitochondrion]	0.10	-
tRNA(Phe), mitchondrial [mitochondrion]	-0.10	-
H+ [extracellular]	-0.16	0.40
phosphate [extracellular]	-	0.25
(S)-malate [extracellular]	-	-0.14
L-serine [extracellular]	-	-0.12
L-glutamine [extracellular]	-	-0.11
ammonium [extracellular]	-	0.11
acetaldehyde [extracellular]	-	-0.10
(S)-lactate [extracellular]	-	-0.10
indol-3-ylacetaldehyde [extracellular]	-	-0.10
guanosine [extracellular]	-	-0.10
(S)-malate [cytoplasm]	-0.22	-
(S)-lactate [cytoplasm]	-0.21	-
ATP [cytoplasm]	-0.20	-
succinate [cytoplasm]	-0.18	-
H+ [cytoplasm]	0.18	-
ADP [cytoplasm]	0.16	-
2-oxoglutarate [cytoplasm]	-0.15	-
TDP [cytoplasm]	-0.12	-
citrate [cytoplasm]	0.10	-
