# Supplementary material for: Principal metabolic flux mode analysis
Source: Bioinformatics. 2018 Feb 6;34(14):2409–17. doi: 10.1093/bioinformatics/bty049 (PMC6041797; doi:10.1093/bioinformatics/bty049)
Supplement: Supplementary Data [file bty049_supp.zip › bty049-suppl_data/PichiaPastorisResultAndAnalysis.zip!/PichiaPastorisResultAndAnalysis/CorrelationPFMandFlux_PichiaPastoris.pdf]

Correlation between PFM and Fluxomic data

Lambda has been optimized using CV

Simulated flux with out noise	Without noise			With 2% Error			With 5% Error			With 10% Error		
	pc1	pc2	pc3	pc1	pc2	pc3	pc1	pc2	pc3	pc1	pc2	pc3
1	-0.16	0.76	0.18	-0.16	0.57	0.75	-0.16	0.57	0.75	-0.16	0.57	0.75
2	0.91	0.16	-0.17	0.91	0.33	0.13	0.91	0.33	0.13	0.91	0.33	0.13
3	0.03	0.46	-0.02	0.02	0.78	0.42	0.02	0.78	0.42	0.02	0.78	0.42
4	-0.20	0.63	0.28	-0.20	0.44	0.63	-0.20	0.44	0.63	-0.20	0.44	0.63
5	0.90	0.15	-0.18	0.90	0.34	0.13	0.90	0.34	0.13	0.90	0.34	0.13
6	0.02	0.43	-0.01	0.01	0.76	0.39	0.01	0.76	0.39	0.01	0.76	0.39
7	-0.10	0.85	0.08	-0.10	0.66	0.83	-0.10	0.66	0.83	-0.10	0.66	0.83
8	0.91	0.16	-0.19	0.91	0.34	0.14	0.91	0.34	0.14	0.91	0.34	0.14
9	0.01	0.34	0.01	0.00	0.68	0.30	0.00	0.68	0.30	0.00	0.68	0.30
10	-0.19	0.67	0.25	-0.19	0.48	0.66	-0.19	0.48	0.66	-0.19	0.48	0.66
11	0.90	0.08	-0.17	0.90	0.29	0.06	0.90	0.29	0.06	0.90	0.29	0.06
12	0.03	0.45	-0.02	0.02	0.77	0.40	0.02	0.77	0.40	0.02	0.77	0.40

Correlation between 3-factor PEMA and Fluxomic data from on *Pichia Pastoris* simulated data

Simulated flux with out noise	Without noise			With 2% Error			With 5% Error			With 10% Error		
1	0.20	0.57	0.80	0.05	0.80	0.57	0.87	0.05	0.57	0.87	0.45	-0.08
2	1.00	-0.06	0.35	0.93	0.55	-0.06	0.30	0.93	-0.06	0.30	0.48	0.91
3	0.34	0.44	0.98	0.23	0.68	0.44	0.76	0.23	0.44	0.76	0.45	0.08
4	0.12	0.77	0.71	0.08	0.62	0.77	0.69	0.08	0.77	0.69	0.62	-0.08
5	1.00	-0.04	0.37	0.93	0.56	-0.04	0.31	0.93	-0.04	0.31	0.49	0.90
6	0.33	0.48	0.98	0.24	0.64	0.48	0.72	0.24	0.48	0.72	0.49	0.08
7	0.25	0.29	0.80	0.00	0.92	0.29	0.97	0.00	0.29	0.97	0.21	-0.07
8	1.00	-0.05	0.36	0.93	0.56	-0.05	0.31	0.93	-0.05	0.31	0.49	0.90
9	0.30	0.55	0.94	0.26	0.56	0.55	0.64	0.26	0.55	0.64	0.55	0.09
10	0.15	0.73	0.74	0.08	0.67	0.73	0.75	0.08	0.73	0.75	0.58	-0.08
11	0.99	0.03	0.35	0.96	0.49	0.03	0.25	0.96	0.03	0.25	0.57	0.91
12	0.33	0.45	0.98	0.23	0.67	0.45	0.75	0.23	0.45	0.75	0.46	0.08

Table 3 : Here we calculate correlation of individual principal fluxes derived from PFMA and PEMA with cleaned fluxomic data. For both model we have considered first 3 principal fluxes. For noise-free fluxomic Data correlations between fluxes or pc's are similar. But with increase of noise in input fluxomic data PEM result deviates a lot and hence resultant principal fluxes remain no more correlated with noise-free fluxes. While the proposed PFMA able to find highly correlated principal fluxes from noisy fluxomic data .

Correlation between PFM and Fluxomic data

With 20% Error

pc1	pc2	pc3
-0.16	0.57	0.76
0.91	0.33	0.19
0.03	0.78	0.48
-0.21	0.44	0.59
0.90	0.34	0.19
0.03	0.76	0.45
-0.09	0.66	0.88
0.90	0.34	0.19
0.02	0.68	0.35
-0.19	0.48	0.64
0.90	0.29	0.11
0.03	0.77	0.47

With 20% Error

0.45	0.25	0.46
0.48	0.09	0.18
0.45	0.51	0.75
0.62	0.35	0.54
0.49	0.09	0.21
0.49	0.54	0.76
0.21	0.14	0.32
0.49	0.07	0.17
0.55	0.56	0.84
0.58	0.32	0.53
0.57	0.12	0.27
0.46	0.52	0.76

A
