# Supplementary material for: Principal metabolic flux mode analysis
Source: Bioinformatics. 2018 Feb 6;34(14):2409–17. doi: 10.1093/bioinformatics/bty049 (PMC6041797; doi:10.1093/bioinformatics/bty049)
Supplement: Supplementary Data [file bty049_supp.zip › bty049-suppl_data/PichiaPastorisResultAndAnalysis.zip!/PichiaPastorisResultAndAnalysis/CorrelationPFMandActiveEMs_PichiaPastoris.pdf]

Correlation between PFM and Active Fluxes for *Pichia Pastoris* simulated data

Lambda has been optimized using LOO cross-validation

Active Ems	Without noise			With 2% Error			With 5% Error			With 10% Error		
	pc1	pc2	pc3	pc1	pc2	pc3	pc1	pc2	pc3	pc1	pc2	pc3
1	0.91	-0.07	-0.09	0.91	0.12	-0.08	0.91	0.12	-0.08	0.91	0.12	-0.08
3	-0.22	0.19	0.47	-0.22	0.01	0.21	-0.22	0.01	0.21	-0.22	0.01	0.21
7	0.92	-0.03	0.11	0.92	0.13	-0.03	0.92	0.13	-0.03	0.92	0.13	-0.03
12	-0.01	-0.07	0.03	-0.02	0.26	-0.10	-0.02	0.26	-0.10	-0.02	0.26	-0.10
13	-0.22	0.10	0.33	-0.22	0.02	0.11	-0.22	0.02	0.11	-0.22	0.02	0.11
14	-0.03	0.02	0.12	-0.04	0.39	0.00	-0.04	0.39	0.00	-0.04	0.39	0.00
16	0.90	-0.09	-0.12	0.90	0.12	-0.11	0.90	0.12	-0.11	0.90	0.12	-0.11
19	0.88	0.25	-0.21	0.88	0.40	0.22	0.88	0.40	0.22	0.88	0.40	0.22
20	0.05	0.66	-0.09	0.04	0.88	0.62	0.04	0.88	0.62	0.04	0.88	0.62
22	0.66	-0.13	-0.10	0.66	0.16	-0.16	0.66	0.16	-0.16	0.66	0.16	-0.16
23	-0.08	0.86	0.05	-0.08	0.67	0.84	-0.08	0.67	0.84	-0.08	0.67	0.84
24	0.05	0.62	-0.07	0.04	0.87	0.57	0.04	0.87	0.57	0.04	0.87	0.57
28	0.90	0.20	-0.20	0.90	0.37	0.18	0.90	0.37	0.18	0.90	0.37	0.18
32	-0.10	0.85	0.09	-0.11	0.66	0.83	-0.11	0.66	0.83	-0.11	0.66	0.83
33	0.04	0.57	-0.05	0.03	0.85	0.53	0.03	0.85	0.53	0.03	0.85	0.53
37	0.91	0.17	-0.19	0.91	0.34	0.14	0.91	0.34	0.14	0.91	0.34	0.14

Correlation between 3-factor PEMA and Active FluxesM from on *Pichia Pastoris* simulated data

Active Ems	Without noise			With 2% Error			With 5% Error			With 10% Error		
	0.92	0.06	0.16	0.98	0.27	0.06	0.01	0.98	0.06	0.01	0.60	0.94
1	-0.09	0.76	0.24	0.06	0.04	0.76	0.10	0.06	0.76	0.10	0.60	-0.03
3	0.89	-0.06	0.08	0.93	0.24	-0.06	-0.03	0.93	-0.06	-0.03	0.48	1.00
7	0.16	0.58	0.62	0.24	0.21	0.58	0.29	0.24	0.58	0.29	0.57	0.07
12	-0.06	1.00	0.34	0.16	0.03	1.00	0.10	0.16	1.00	0.10	0.83	-0.06
13	0.14	0.80	0.65	0.31	0.11	0.80	0.20	0.31	0.80	0.20	0.76	0.09
14	0.92	0.16	0.20	1.00	0.26	0.16	0.01	1.00	0.16	0.01	0.69	0.93
16	0.99	-0.12	0.40	0.87	0.64	-0.12	0.40	0.87	-0.12	0.40	0.40	0.86
19	0.38	0.16	0.98	0.14	0.85	0.16	0.92	0.14	0.16	0.92	0.20	0.06
20	0.76	0.31	0.42	0.82	0.33	0.31	0.17	0.82	0.31	0.17	0.70	0.70
22	0.26	0.19	0.78	-0.02	0.94	0.19	0.99	-0.02	0.19	0.99	0.13	-0.06
23	0.37	0.25	0.99	0.17	0.81	0.25	0.88	0.17	0.25	0.88	0.28	0.07
24	1.00	-0.09	0.38	0.90	0.60	-0.09	0.35	0.90	-0.09	0.35	0.45	0.88
28	0.25	0.31	0.80	0.00	0.91	0.31	0.97	0.00	0.31	0.97	0.23	-0.07
32	0.36	0.34	1.00	0.20	0.76	0.34	0.84	0.20	0.34	0.84	0.36	0.08
33	1.00	-0.06	0.36	0.92	0.56	-0.06	0.31	0.92	-0.06	0.31	0.48	0.89
37												

Table 2 : Here we calculate correlation of individual principal fluxes derived from PFMA and PEMA with active Ems. For both model we have considered first 3 principal fluxes. For noise-free fluxomic data correlations between active EM or pc's are similar. But with increase of noise in input fluxomic data PEMA result deviates a lot and hence resultant principal fluxes remain no more correlated with Active Ems. While the proposed PFMA able to find highly correlated principal fluxes from noisy fluxomic data .

Correlation between PFM and Active Fluxes

With 20% Error

pc1	pc2	pc3
0.90	0.12	-0.07
-0.23	0.01	0.10
0.91	0.13	-0.04
0.00	0.26	-0.07
-0.23	0.02	0.02
-0.04	0.39	-0.03
0.89	0.12	-0.10
0.88	0.40	0.29
0.06	0.88	0.71
0.67	0.16	-0.12
-0.07	0.67	0.90
0.05	0.87	0.66
0.89	0.37	0.24
-0.10	0.66	0.87
0.04	0.85	0.60
0.90	0.34	0.20

With 20% Error

0.60	0.14	0.17
0.60	0.43	0.40
0.48	0.25	0.07
0.57	0.49	1.00
0.83	0.40	0.58
0.76	0.63	0.79
0.69	0.14	0.24
0.40	0.04	0.13
0.20	0.36	0.50
0.70	0.29	0.72
0.13	0.11	0.27
0.28	0.41	0.56
0.45	0.05	0.15
0.23	0.15	0.33
0.36	0.46	0.62
0.48	0.06	0.16
